# Understanding the Relationship between Rainstorm-Related Experiences and PTSD among Chinese Adolescents after Rainstorm Disaster: The Roles of Rumination and Social Support

**DOI:** 10.3389/fpsyg.2016.01407

**Published:** 2016-09-15

**Authors:** Rui Zhen, Lijuan Quan, Benxian Yao, Xiao Zhou

**Affiliations:** ^1^Beijing Key Laboratory of Applied Experimental Psychology, School of Psychology, Beijing Normal University, BeijingChina; ^2^College of Educational Science, Anhui Normal University, WuhuChina; ^3^I-Core Research Center for Mass Trauma, Bob Shapell School of Social Work, Tel Aviv University, Tel AvivIsrael

**Keywords:** adolescents, rainstorm-related experiences, rumination, social support, PTSD

## Abstract

Posttraumatic stress disorder (PTSD) is prevalent among adolescents following natural disasters, and the trauma experiences represent a critical risk factor for PTSD. Nevertheless, the underlying mechanism of adolescents’ PTSD following trauma experiences remains unclear. Rumination appears to be a mediating factor between trauma experiences and PTSD, and social support may moderate this mediating relationship between trauma experiences, rumination, and PTSD, but few studies have examined these assumptions. Thus, this study aimed to assess the mediating role of rumination and the moderating role of social support in the relationship between rainstorm-related experiences and PTSD among adolescents, following a rainstorm in China. Nine hundred and fifty-one middle school students completed self-report questionnaires, and structural equation modeling was conducted to examine the potential moderated mediation effect. Rainstorm-related experiences had a direct and positive effect on PTSD, and also indirectly influenced PTSD via rumination. Moreover, social support work to buffer the direct effect of rainstorm-related experiences on PTSD, but not the effect of rumination on PTSD. Implications for clinical practice and research are discussed along with study limitations.

## Introduction

Posttraumatic stress disorder (PTSD) is relatively prevalent, with lifetime estimates in general samples ranging from 7 to 12% (Breslau, 2001). The prevalence of PTSD in traumatized populations following natural disasters ranges from 5 to 60% (Galea et al., 2005). More specifically, some studies indicate that 6.5~82.6% of traumatized people in China report PTSD following natural disasters (Cao et al., 2015). Adolescent survivors may experience more severe impact due to their susceptibility to trauma (Eth, 2008), such that available studies on adolescents after natural disasters show that the prevalence of PTSD in such individuals amounts to 95% (Saigh et al., 1999). Particularly, the prevalence of PTSD after rainstorms among Chinese middle students reached 10.57%, which implied that people’s mental health following rainstorms needed more attention (Zeng et al., 2011).

The prevalence of PTSD among adolescents after natural disasters may be critically attributed to the degree of trauma exposure (Verger et al., 2000; Liu et al., 2006; Bokszczanin, 2007). The risk factors model of trauma (Freedy et al., 1992) suggests that trauma experiences are the primary risk factors for PTSD, although many factors can elicit PTSD. The shattered world assumption (Janoff-Bulman, 2010) proposes that trauma experiences can shatter people’s core beliefs and assumptions about the self, others, and the world, which can lead people to develop highly negative assumptions and associated negative emotions, culminating in the occurrence of PTSD. In addition, studies of adolescents after natural disasters have found that compared to those without trauma experiences, more adolescents with trauma experiences report PTSD (Fan et al., 2011).

Janoff-Bulman (2010) also claimed that after trauma experiences, traumatized people’s internal disequilibrium before and after traumatic experiences could lead them to become immersed in the traumatic event, such that they think repeatedly on it. This repetitive thought has been dubbed rumination (Nolen-Hoeksema, 2000; Nolen-Hoeksema et al., 2008), which refers to a mode of responding to distress that involves repetitively and passively focusing on symptoms of distress and on the possible causes and consequences of these symptoms. Rumination on traumatic events throws the focus of traumatized people onto negative trauma outcomes, and in turn increases negative emotion and cognition (Blackburn and Owens, 2016), which finally results in PTSD (Chuen Yee Lo et al., 2012; Borders et al., 2015; Roley et al., 2015). Thus, it is likely that trauma experiences predict PTSD because they elicit rumination.

The potential influence of trauma experiences on PTSD via rumination may be changed; nevertheless, few have discussed the circumstances that might influence this relationship (Carpenter et al., 2010). As Lepore (2001) outlines in his social-cognitive theory, social support is crucial in perpetuating or breaking the pathway from trauma experiences to PTSD via rumination. For instance, a supportive environment can facilitate people’s disclosure around trauma experiences. This promotes an open discussion about the trauma and related clues with others (Zhou et al., 2014b), and provides opportunities for others to understand their trauma experiences and to help with the processing of trauma clues as well as negative emotions (Monk and Nelson Goff, 2014). Therefore, although rumination increases attention on negative aspects of the trauma experience, the derived negative emotions from these thoughts should be ameliorated by others’ understanding and help provided in a supportive environment, and thus PTSD will be also eased. In contrast, it is hard for people to process and regulate rumination-elicited negative emotions when they lack support (Nelson Goff et al., 2015). That is, social support may moderate the relationship between rumination and PTSD.

Moreover, social support may also moderate a direct relationship between trauma experiences and PTSD. Here, Cohen and Syme (1985) proposed a buffering model of social support to characterize the process through which social support has beneficial effects on psychological outcomes during or after stress (Carpenter et al., 2010). A supportive environment should encourage traumatic survivors to redefine the threat from the stressful event and to create meaning in the world, thereby possibly reducing the negative effect of trauma experiences on psychological reactions (Zhou et al., 2014b). However, a non-supportive environment can increase survivors’ negative reactions to trauma reminders, which limits them in terms of reframing trauma experiences (Lepore and Greenberg, 2002; Carpenter et al., 2010). As a result, the effect of trauma experiences on psychological reactions may be exacerbated. Thus, we made the assumption that social support would moderate a direct relationship between trauma experiences and PTSD.

Although the relationships described above make theoretical sense, few studies have assessed assess the mediating and/or moderating roles of rumination and social support in the relationship between trauma experiences and PTSD, particularly among adolescents, who are experiencing rapid development of cognition and emotion and are susceptible to trauma impact (Tedeschi and Calhoun, 2004). Rainstorm disasters are common in the middle-lower reaches of the Yangtze and Huai Rivers in China every year, which likely result in many negative psychological outcomes for adolescents affected by such disasters. The present study sought to elucidate the mechanisms by which rainstorm-related experiences might produce PTSD in adolescents, with a specific focus on the potential exacerbating or buffering roles of rumination and social support.

Given this backdrop, and based on the shattered world assumption theory (Janoff-Bulman, 2010) as well as the buffering theory of social support (Cohen and Syme, 1985; Lepore, 2001), we proposed the following hypotheses: (1) Rumination will mediate the relationship between rainstorm-related experiences and PTSD, and (2) social support will moderate the pathway from rainstorm-related experiences to rumination and then PTSD.

## Materials and Methods

### Participants and Procedures

This study was approved by the Research Ethics Committees of the authors’ affiliated institutions and was conducted with the permission of the principals of the participating schools. To recruit participants, we first chose the areas where rainstorms occur frequently in China, with total rain amounting to more than 200 mm during 24 h, according to the China Meteorological Administration. We then selected several cities such as Nanjing, Huainan, and Huangshan, located in the middle-lower reaches of the Yangtze River and Huai River in China, the areas that have a relatively high occurrence of rainstorm disasters. In fact, the cities we selected are experiencing severe rainstorms in recent years, and these rainstorms have caused widespread loss of life and property and led people to psychological distress. Particularly, several strong rainstorms occurred in the cities from early June to late July 2015, wherein many schools suffered from severe damage, and students were asked to stay at home. As adolescents might be affected because of their susceptibility to trauma from natural disasters (Margolin et al., 2010), we chose them as our study subjects. Then, we started to carry out the investigation in September 2015, and finished it in October 2015, about 2 months after the latest rainstorm, the time when the new semester started and students were available to participant in the research. Classes in the participating schools were randomly selected and everyone who attended these classes on the date of the survey was recruited to participate. There were no exclusion criteria. Compensation was not provided. The purpose of the study and the voluntary nature of students’ participation were highlighted before the survey, and written consent forms that emphasized the right of each participant to withdraw from the survey at any time were provided to participants. The researchers administered the questionnaire packets in a classroom setting without teachers present. The participants first provided demographic information that included gender and age. They were then asked to complete measures that assessed rainstorm-related experiences, rumination, social support, and PTSD. After the questionnaire packets were completed, the participants were told that school psychologists or teachers were available to provide any psychological/counseling services that they might need. Nine hundred and fifty-one middle school students participated in this investigation. Four hundred and fifty-three (47.6%) were male and 490 (51.6%) were female, and eight (0.8%) participants did not report gender. Mean participant age was 14.78 (*SD* = 1.70) years old, with a range from 12.0 to 19.0.

### Measures

#### Rainstorm-Related Experiences

We used our own self-developed questionnaire to assess adolescents’ rainstorm-related experiences. There is no well-established measure of rainstorm-related experiences, so we developed one based on the development of the Hurricanes-related Traumatic Experiences Questionnaire (Vernberg et al., 1996). We also carried out qualitative interviews with some adolescents who were severely affected by rainstorms. These interviews and additional input from psychologists helped us to develop the questionnaire items. Seven items constituted the formal questionnaire (e.g., *our house was damaged by the rainstorm*), and were each rated on a 5-point Likert scale ranging from 0 (*completely disagree*) to 4 (*completely agree*). Each student evaluated their exposure to daily life-threatening experiences or to disruption/loss in the week following the latest rainstorm. The questionnaire had good internal consistency (α = 0.83).

### Rumination

Rumination was assessed using the Cognitive Emotion Regulation Questionnaire (revised Chinese version; Zhu et al., 2007). This measure is a 36-item instrument designed to measure the following cognitive coping strategies: Self-blame, Acceptance, Rumination, Positive Refocusing, Refocus on Planning, Positive Reappraisal, Putting into Perspective, Catastrophizing, and Other-blame (Garnefski et al., 2004). All items are rated on a 5-point Likert scale that ranges from 0 (*completely disagree*) to 4 (*completely agree*). This questionnaire shows good reliability in Chinese samples (Zhu et al., 2007). We only used the rumination subscale here, and the internal consistency of this subscale was acceptable here (α = 0.71).

### Social Support

Social support was assessed using the Social Net Questionnaire (Zou, 1999). This questionnaire is a 20-item scale designed to measure the following forms of perceived social support: Emotive support, instrumental support, companionship, affirmative evaluation, and intimacy. All items are rated on a 5-point scale ranging from 0 (*completely disagree*) to 4 (*completely agree*). The modified scale has exhibited good reliability and construct validity among adolescent survivors of natural disasters (Zhou et al., 2014a). In this study, the internal consistency of the inventory was good (α = 0.95).

### PTSD

Posttraumatic stress disorder was assessed using the PTSD Checklist for DSM-5 (Weathers, 2013). This measure is a 20-item self-report scale that was designed to assess the occurrence and frequency of PTSD symptoms in relation to the most distressing event experienced by an individual. All of the items were translated into Chinese, and the respondents rated symptom frequencies during the last 2 weeks on a 4-point Likert scale ranging from 0 (*not at all/only once*) to 3 (*almost always or more than five times a week)*. There are four subscales: Re-experiencing, negative cognition and emotion alteration, avoidance, and hyper-arousal. An overall severity score is generated by summing the scores for the four symptom types. This scale shows good reliability and validity in adolescent samples after earthquake (Wang et al., 2015). The scale demonstrated good internal consistency for the present sample (α = 0.90).

### Data Analysis Strategies

Descriptive analyses were conducted for all of the measures administered. Correlations were calculated to examine the associations between major variables. Statistical analyses were conducted using AMOS 17.0 software (Arbuckle, 2006). Before statistical analyses, Little’s Missing Completely at Random (MCAR) test results revealed that the data were missing at random (MAR, χ^2^(18) = 22.31, *p* = 0.218), and we therefore adopted maximum likelihood estimates (ML) to handle missing data.

In addition, we examined the distribution of scale scores, and found that Skewness and Kurtosis were 1.15 and 1.24 for rainstorm-related experiences, -0.18 and -0.07 for rumination, -0.39 and 0.04 for social support, and 0.84 and 0.80 for PTSD. These findings indicate that the scores do not substantially deviate from a normal distribution. ML based on raw score correlations was thus used in the model. To evaluate model fit, we used chi-square values, the comparative fit index (CFI), Tucker-Lewis index (TLI), normed fit index (NFI), and the root mean square error of approximation (RMSEA). A non-significant chi-square value indicates a good fit between the model and data. The general model acceptance cutoffs for NFI, CFI, and TLI are equal to or greater than 0.90, along with an RMSEA of equal to or less than 0.08 (Wen et al., 2004).

**FIGURE 1:**
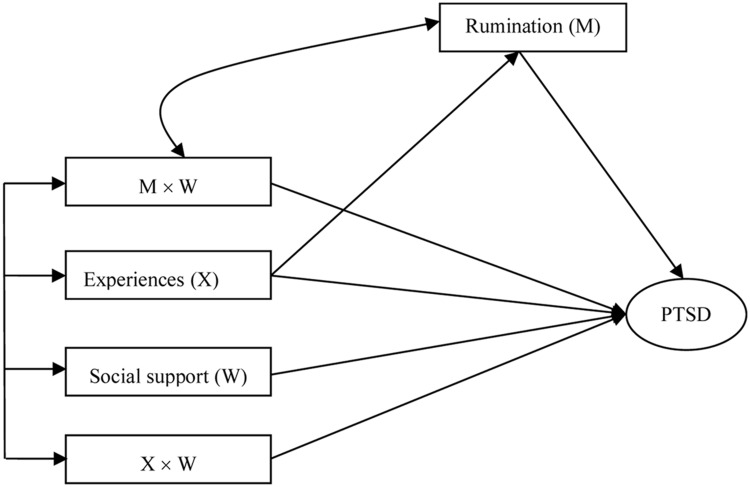
**Assumptive model of moderated mediation effect.** Experiences, Rainstorm-related experiences.

In the moderated mediation examination, all independent variables were first centered on their respective means to reduce multicollinearity between main effects and interaction terms, and to increase the interpretability of the coefficients on interaction terms (Cohen et al., 2013). We then followed the procedures recommended by Preacher et al. (2007) and Hayes (2013), and built a structure equation model (**Figure 1**). In this model, rainstorm-related experience directly predicts PTSD but also exerts an indirect effect via rumination. Social support predicts PTSD, as does the interaction term between rainstorm-related experiences and social support. Finally, the interaction term between rumination and social support predicts PTSD.

Next, to test the significance of the indirect effect of rainstorm-related experiences on PTSD via rumination, we conducted a bias-corrected bootstrap test with a 95% confidence interval, constructing 5000 bootstrap samples. We also used simple slope tests to further examine the significance of the interaction terms.

## Results

### Descriptive Statistics and Correlations among Main Measures

**Table 1** shows descriptive statistics and correlations among main measures. The mean levels of rainstorm-related experiences, rumination, social support, and PTSD were 4.33, 9.49, 47.88, and 17.30, respectively. Correlation analyses indicated that gender was significantly associated with rainstorm-related experiences and social support, and that age was significantly associated with social support. Except for the relationship between rainstorm-related experiences and social support, all the relationships among the main variables were positive and significant. We also performed the present analyses controlling for age and gender. Results did not differ across controlled and uncontrolled analyses, suggesting that these demographic variables had no virtually impact on the associations among main variables. Thus, we did not control for gender and age in the analyses reported here.

**Table 1:** Means, standard deviations, and correlations between rainstorm-related experience, rumination, social support, and PTSD (posttraumatic stress disorder).

	*M* ±*SD*	1	2	3	4	5	6
1. Gender	–	–					
2. Age	14.78 ± 1.7	0.04	–				
3. Rainstorm-related experience,	4.33 ± 4.25	0.14***	0.05	–	0.07*	-0.02	0.14***
4. Rumination	9.49 ± 3.38	-0.01	0.04	0.07*	–	0.21***	0.27***
5. Social support	47.88 ± 17.12	0.17***	-0.10**	0.001	0.20***	–	0.10**
6. PTSD	17.30 ± 10.41	-0.02	-0.02	0.14***	0.28***	0.10**	–

The numbers to the lower left are correlation coefficients without controlling for age and gender, the numbers to the top right are correlation coefficients after controlling for age and gender.

***p < 0.001, **p < 0.01, *p < 0.05.

### Moderated Mediation Effect

**FIGURE 2:**
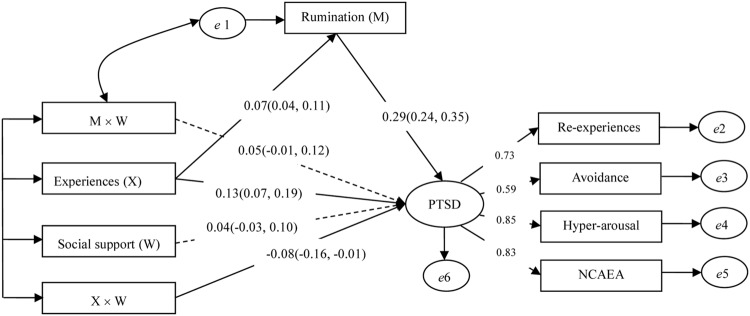
**Final model of moderated mediation effect.** Solid lines indicate that the predictive coefficients are significant at the 0.05 level; dotted lines indicate that the predictive coefficients are non-significant at the 0.05 level. ‘*e*’ indicates errors. Experiences, Rainstorm-related experiences; Re-experiencing, Re-experiencing symptoms of PTSD (posttraumatic stress disorder); Avoidance, Avoidance symptoms of PTSD; Hyper-arousal, Hyper-arousal symptoms of PTSD; NCAEA, Negative cognition and emotion alteration associated with PTSD. The numbers in brackets indicate 95% confidence intervals.

We conducted analysis of the model displayed in **Figure 1**, and found that this model fit the data well (χ^2^/df = 3.589, CFI = 0.975, TLI = 0.950, NFI = 0.966, RMSEA = 0.052). Final path analysis results are presented in **Figure 2**. As seen in **Figure 2**, rainstorm-related experiences were a significant positive predictor of both PTSD and rumination, and rumination had a significant effect on PTSD. This suggests that rumination plays a mediating role in the relationship between rainstorm-related experiences and PTSD. Additionally, social support was a non-significant predictor of PTSD, although the interaction between rainstorm-related experiences and social support significantly and negatively predicted PTSD, which indicates that social support moderated the association between rainstorm-related experiences and PTSD. Moreover, the interaction between rumination and social support was also a non-significant predictor of PTSD, suggesting that social support does not moderate the association between rumination and PTSD. Taken together, our results indicated that rumination mediates and social support moderates the relationship between rainstorm-related experiences and PTSD.

**FIGURE 3:**
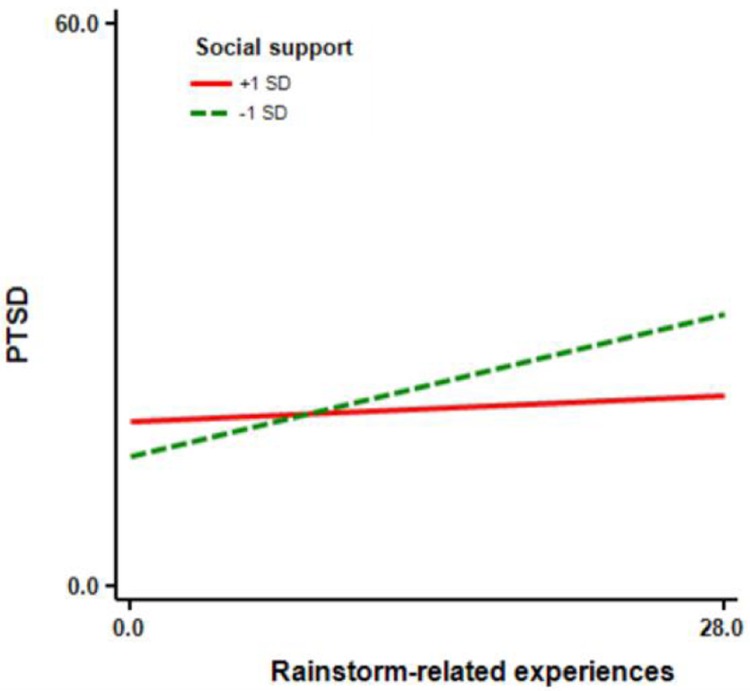
**Relationship between rainstorm-related experiences and PTSD at different levels of social support**.

We conducted a bias-corrected bootstrap test with a 95% confidence interval to further test mediator significance. The mediation effect size was 0.02, and the 95% CI for the indirect path ranged from 0.002 to 0.041, indicating that rumination did in fact mediate the relationship between rainstorm-related experiences and PTSD, according to Preacher and Hayes’ (2008) guidelines. Similarly, we used a simple slope test to further examine whether the moderating effect of social support was significant. We graphed the relationship between rumination and PTSD for participants whose levels of social support were 1 SD above or below the mean (**Figure 3**).

For participants 1 SD above the social support mean, increased rumination was not significantly associated with changes in PTSD (*simple slope* = 0.10, *t* = 0.86, *p* = 0.387). In contrast, for participants 1 SD below the mean, increased rumination was associated with a significant increase in PTSD symptoms (*simple slope* = 0.54, *t* = 4.84, *p* < 0.001).

## Discussion

To our knowledge, the present study is first to examine the mediating role of rumination and moderating role of social support in the relationship between rainstorm-related experiences and PTSD, among adolescents after rainstorms in China. The findings indicated that rainstorm-related experiences work to directly increase PTSD, but also play an indirect role by eliciting more rumination. More importantly, the results suggested that although social support has no significant moderating effect on the indirect path from rumination to PTSD, support did moderate the direct relationship between rainstorm-related experiences and PTSD.

Consistent with previous studies (Bokszczanin, 2007; Chen et al., 2015; Dixon et al., 2015), our findings suggest that rainstorm-related experiences can directly lead to PTSD. This supports the shattered world assumption (Janoff-Bulman, 2010), which proposes that traumatic experiences can challenge people’s stable belief systems such that more negative cognition and emotion emerges (Janoff-Bulman, 1989), and in turn more PTSD symptoms are elicited.

Furthermore, shattered world assumption theory also emphasizes the vital role that negative cognitive activities such as rumination may play in the occurrence of PTSD (Janoff-Bulman, 2010). Consistent with this, our study found that rumination mediates the association between rainstorm-related experiences and PTSD. Trauma can shatter people’s stable world views of the self, others, and the world, which propels them to rebuild belief systems following trauma. During this process, repetitive and passive thoughts get activated, namely, rumination (Janoff-Bulman, 2010). It is thought that rumination can exacerbate and prolong distress through the following mechanisms (Nolen-Hoeksema, 1991). First, rumination can enhance the effect of negative mood on thinking, such that traumatized people are more likely to use negative thoughts and memories inspired by negative mood to understand their current circumstances. Second, rumination can interfere with the process of effective problem-solving, in part by making people think more pessimistically and fatalistically (Nolen-Hoeksema et al., 2008). These will lead traumatized people to experience more negative cognition and emotion, and in turn elicit PTSD symptoms (Egan et al., 2014; Hu et al., 2014).

Nevertheless, the mediation effect size of rumination was relatively small. One possible explanation could be the small effect size of rain-related experiences on rumination. The frequency of rainstorms experienced by the present sample may have encouraged the development of resilience (Schneier et al., 2012), would could buffer against the use of rumination (Tugade and Fredrickson, 2004).

An interesting finding is that social support may not in fact play a significant role in PTSD. One possible explanation for this finding is that if constant social support from others is provided even 2 months after trauma, adolescents may be precluded from learning how to use their own coping skills, which will further damage their self-efficacy and enthusiasm in coping with trauma (e.g., Zhou et al., 2014b). Under such circumstances, social support might be expected to actually hinder recovery.

Inconsistent with our hypothesis, we did not find a moderating effect of social support on the relationship between rumination and PTSD. This result also diverges from the assumption of Lepore (2001), who outlines that social support can intervene during the development of PTSD following trauma. This may be due to the nature of rumination. Adolescents who ruminate frequently are more likely to express dissatisfaction with their social support networks (Flynn et al., 2010), and may engage in co-rumination (i.e., excessive, repetitive discussion of problems) during social interactions, which will in turn precipitate or exacerbate symptoms of anxiety (Vélez et al., 2015). PTSD would be unlikely to remit under such circumstances.

Our findings did indicate that social support moderates a direct effect of rainstorm-related experiences on PTSD, and this is consistent with our hypothesis as well as supporting the buffering model of social support (Cohen and Syme, 1985). Specifically, for those adolescents with low social support, rainstorm-related experiences were positively and significantly related to PTSD, whereas for those with high social support, the relationship between rainstorm-related experiences and PTSD was non-significant. According to the buffering model, social support can provide necessary resources for adolescent survivors to cope with rainstorm-related experiences and negative mental reactions (Cohen and Syme, 1985), thereby promoting coping self-efficacy (Zhou et al., 2016). Additionally, social support can provide safe surroundings that create feelings of security and belonging (Zhou et al., 2014a), in which adolescents tend to experience more positive emotions (Zhou et al., 2016). Therefore, social support can buffer the effects of rainstorm-related experiences on PTSD.

Several design and measurement limitations must be acknowledged. First, the participants were not randomly selected for convenience of investigation, such that sampling bias might exist. Second, all variables were measured using self-report scales, such that associations between measures might be affected by common-method variance. Third, though this study examined a significant role of rumination in the relation between rainstorm-related experience and PTSD, the effect size was relatively small, thus future study can further examine this mediation effect in other samples after rainstorms. Moreover, except for gender and age, this study did not take other socio-demographic characteristics (e.g., socioeconomic status, parental education, etc.) into consideration, although such variables may also play a role in PTSD. Additionally, this study was conducted using a sample of adolescents after rainstorms in China, so generalizations to other people with other trauma-related experiences must be done with caution.

Despite these limitations, this study contributes new knowledge regarding the relationship between trauma experiences and PTSD. In particular, this study found that rumination mediated and social support moderated the relationship between rainstorm-related experiences and PTSD. From an intervention and health-enhancement perspective, this study also highlights important implications for adolescents after rainstorms. Clinical efforts should focus on decreasing rumination, and helping adolescents to shift their attention away from the negative aspects of rainstorms. Additionally, school psychologists or parents should provide emotional and material support for adolescents and work to foster a generally supportive environment. Additionally, encouraging adolescents to acknowledge trauma cues and negative emotions may mitigate the negative effect of natural disasters.

## Author Contributions

RZ and XZ contributed the design of this study, analysis of data, and the writing and revision of the manuscript; LQ and XZ contributed the collection of data.

## Conflict of Interest Statement

The authors declare that the research was conducted in the absence of any commercial or financial relationships that could be construed as a potential conflict of interest.
